# Antibacterial and Antioxidant Activity of *Dysphania ambrosioides* (L.) Mosyakin and Clemants Essential Oils: Experimental and Computational Approaches

**DOI:** 10.3390/antibiotics11040482

**Published:** 2022-04-05

**Authors:** Fahd Kandsi, Amine Elbouzidi, Fatima Zahra Lafdil, Nada Meskali, Ali Azghar, Mohamed Addi, Christophe Hano, Adil Maleb, Nadia Gseyra

**Affiliations:** 1 Laboratory of Bioresources, Biotechnology, Ethnopharmacology and Health, Faculty of Sciences, Mohammed First University, B.P. 717, Oujda 60000, Morocco; kandsifahd1994@gmail.com (F.K.); lafdil.fatimazahra@ump.ac.ma (F.Z.L.); ngseyra@hotmail.com (N.G.); 2 Laboratoire d’Amélioration des Productions Agricoles, Biotechnologie et Environnement (LAPABE), Faculté des Sciences, Université Mohammed Premier, Oujda 60000, Morocco; amine.elbouzidi@ump.ac.ma (A.E.); nada.meskali@gmail.com (N.M.); 3 Laboratoire de Microbiologie, Centre Hospitalier Universitaire (CHU), Oujda 60000, Morocco; a.azghar@ump.ac.ma (A.A.); a.maleb@ump.ac.ma (A.M.); 4 Laboratoire de Biologie des Ligneux et des Grandes Cultures, INRAE USC1328, University of Orleans, CEDEX 2, 45067 Orléans, France; 5 Le StudiumInstitue for Advanced Studies, 1 Rue Dupanloup, 45000 Orléans, France

**Keywords:** *Dysphania ambrosioides*, antioxidant activity, antimicrobial activity, essential oils, computational study, molecular docking

## Abstract

*Dysphania ambrosioides* (L.) Mosyakin and Clemants, also known as Mexican tea, and locally known as Mkhinza, is a polymorphic annual and perennial herb, and it is widely used in folk medicine to treat a broad range of illnesses in Morocco. The aim of this study was to determine the phytochemical content and the antioxidant and the antibacterial properties of essential oils isolated from *D. ambrosioides* aerial components, growing in Eastern Morocco (Figuig). Hydrodistillation was used to separate *D. ambrosioides* essential oils, and the abundance of each phytocompound was determined by using Gas Chromatography coupled with Mass Spectrometry (GC–MS). In vitro 2,2-diphenyl-1-picrylhydrazyl (DPPH) radical scavenging assay and inhibition of β-carotene/linoleic acid bleaching assays were used to determine *D. ambrosioides* essential oils’ antioxidant activity. The findings revealed relative antioxidative power and modest radical scavenging. The antibacterial activity of the essential oils was broad-spectrum, with *Escherichia coli*, *Staphylococcus aureus*, and *Enterococcus faecalis* as the most susceptible strains tested. To elucidate the physicochemical nature, drug-likeness, and the antioxidant and antibacterial action of the identified phytocomponents, computational techniques, such as ADMET analysis, and molecular docking were used.

## 1. Introduction

As a result of the indiscriminate use of antibiotics and the rapid dissemination of life-threatening multidrug-resistant (MDR) bacterial strains, the development of novel yet effective antimicrobial compounds, as well as new feasible alternatives employing natural products to minimize the development of this threat for resistant pathogens, is of a crucial importance [1]. Several investigations have demonstrated the beneficial use of natural substances as antimicrobial agents against MDR bacteria [2]. Plant-derived metabolites, such as essential oils (EOs), have been intensively researched for their prospective use as antibacterial agents, such as lavender, oregano, clove, etc. [3,4,5,6]. However, antibacterial mechanisms of action are linked to many properties of Eos, such as hydrophobicity, which results in enhanced cell permeability and subsequent leaking of cell contents, and also might be recognized as cell growth inhibition or cell death [7]. Burt et al. [8] were the first to report on the effect of EO components on protein synthesis, among other antibacterial mechanisms previously reported in the literature, of which we cite pH disturbance by impairing the pH homeostasis in the bacterial wall, leading the membrane to lose its capacity to block protons [9], the effect of EOs on bacterial DNA [10], etc.

EOs are naturally volatile and complex compounds that are produced as secondary metabolites by aromatic plants, typically obtained by steam or hydro-distillation. EOs have been utilized since time immemorial for their antioxidant, allelopathic, insecticidal, acaricidal [11,12], larvicidal [13], antibacterial, antifungal [14], and even anticancer activities [15]. Synthetized in complex secretory structures, such as glands, secretory cavities, and resin conduits, via the malonic acid, mevalonate, and methyl-d-erythritol-4-phosphate pathways (MEP), EOs comprise a variety of chemicals, such as terpenes, terpenoids, phenylpropenes, and phenolics, and may also contain fatty acids, oxides, alcohols, esters, aldehydes, ketones, and sulfur derivatives [16,17]. These compounds contribute to the distinct and often unique aromatic and bioactive capabilities of a variety of herbs and spices [18].

Eastern Morocco is characterized by its large biodiversity pertaining to medicinal herbs [19], which gained considerable interest in pharmacological studies, leading to drug development [20]. *Dysphania ambrosioides* (L.) Mosyakin and Clemants, locally known as M’khinza, named erstwhile *Chenopodium ambrosioides*, is native to South America, Central America, and Mexico [21,22]. It is an aromatic annual or perennial herb covered with hair secreting oils [23,24]. The species is widely cultivated in sub-tropical and sub-temperate regions devoted to consumption as a leafy vegetable [25,26]. Due to its spread all over Morocco, *D. ambrosioides* has gained a major consideration in traditional medicine [27,28], displaying potency against renal disorders [29], as well as possessing antidiabetic [30], antispasmodic [21], anti-inflammatory [31], and antimalarial properties [32].

Taking the preceding facts into consideration, the purpose of the research reported here was to investigate the phytochemical profile and to emphasize the antibacterial and antioxidant activities of *D. ambrosioides* essential oils (DAEOs) extracted from various parts of the plant (stem, leaves, and flowers). Parallel to the experimental results, computational studies, specifically the ADMET (absorption, distribution, metabolism, excretion, and toxicity) analysis, which was used to forecast DAEOs’ drug-likeness and evaluate their various pharmacokinetic parameters, were also carried out in order to uncover the processes underlying the observed effects. Protein target-based antioxidant and antibacterial mechanisms were predicted by using molecular docking.

## 2. Results and Discussion

### 2.1. Phytochemical Profile of DAEOs Analyzed by Gas Chromatography–Mass Spectrometry

The extraction yields of *D. ambrosioides* essential oils were as follows: 4.20% for leaves and 11.74% for flowers, and the highest yield was found in stems with 12.30%. The phytochemical analysis carried out with GC–MS revealed a richness of monoterpenoids in *D. ambrosioides* essential oils (Supplementary Figure S1).

**Figure 1:**
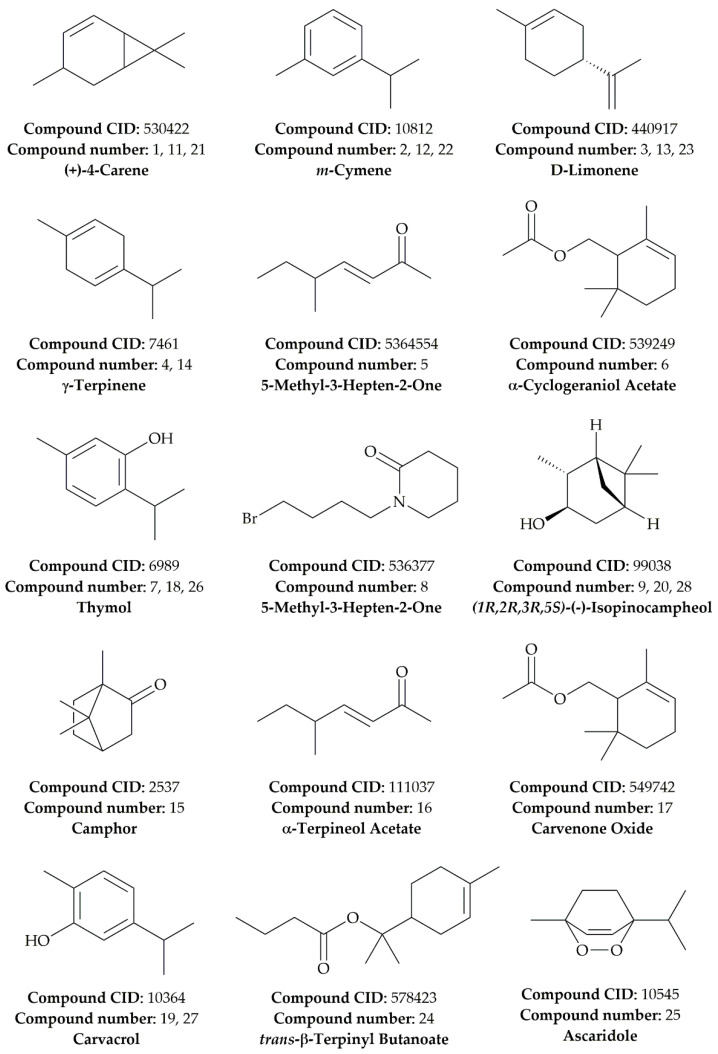
Chemical structures of the identified phytocompounds by GC–MS in *D. ambrosioides* essential oils, along with their compound CID.

The essential oils’ phytochemical profile varies between each part of the species, revealing 15 compounds overall (Figure 1). The most abundant compounds in stem essential oil (SEO) are (+)-4-Carene (50.5%), α-Cyclogeraniol Acetate (22.64%), and *(1R,2R,3R,5S)*-(-)-Isopinocampheol (8.87%), respectively. As for SEO, leaves essential oil (LEO), (+)-4-Carene (46.2%) is the main compound, followed by *m*-Cymene (20.74%) andα-Terpineol Acetate (14.5%). Moreover, *trans*-β-Terpinyl Butanoate is shown to be the major phytoconstituent in flowers essential oil (FEO), with 31.13%, followed by (+)-4-Carene (28.05%) and *(1R,2R,3R,5S)*-(-)-Isopinocampheol (18.06%). The presence of the molecules identified in DAEOs (Table 1) was also reported in previous research [33,34,35,36]. The scented aroma of *D. ambrosioides* EO is linked primarily to Ascaridole, which is one of its major constituents [37,38,39]. However, in the present study, Ascaridole was determined only in a low percentage (1.16%). The present finding is in adequation with de Lacerda Neto et al. (2021), who have found a percentage of 1.48% [36].

### 2.2. Molecular Docking Results

#### Prediction of a Protein Target-Based Antioxidant and Antibacterial Mechanisms In Silico

Molecular docking is a potent computational approach that is frequently utilized to acquire important insight into the molecular processes of pharmacologically active drugs. In this study, molecular docking was used to uncover a putative mechanism of action associated with the antioxidant and antibacterial properties of DAEOs components. The observed results, in the form of binding affinity values, may indicate an increased/decreased affinity of the studied molecule toward the given target in comparison to a native ligand (a known inhibitor), assuming that binding energy reduces as compound affinity increases. This method was used to assess the binding affinity of the 15 essential oil compounds to target proteins known to have bactericidal/bacteriostatic actions, including DNA Gyrase Topoisomerase II, Enoyl-Acyl Carrier Protein Reductase, and Glucosamine-6-phosphate synthase (PDB IDs: 1KZN, 3GNS, and 2VF5, respectively) [40,41,42,43]. The reduction of ROS production in vitro by DAEOs compounds was mechanistically evaluated by assessing their molecular docking contacts with certain enzymatic proteins, namely Lipoxygenase-3 (PDB ID: 1N8Q) [44], Cytochrome P450 (PDB ID: 1OG5) [45], NADPH oxidase (PDB ID: 2CDU) [46], and Bovine Serum Albumin (PDB ID: 4JK4) [47]; all of these proteins have long been described as target receptors for antioxidant chemicals and are recognized to have a part in the process of oxidative homeostasis in the body.

A heat-map-type table using a red–yellow–green three-color scheme, ranging from the lowest energy values, highlighted in red (in most cases corresponding to the docking score of the native ligand), to the highest, highlighted in green, was used to present docking scores (Table 2) in order to easily discover a group comprising chemicals that have a tendency to function as potential inhibitors by comparing their lowest values to that of the native ligand for a specific protein.

Lipoxygenases are a family of metal-containing enzymes that catalyzes lipid peroxidation of poly-unsaturated free fatty acid via a redox mechanism in which the transformation of the active site iron Fe^2+^ to Fe^3+^ leads to the generation of oxygen-centered fatty acid hydroperoxide radical, leading to various pathogenic diseases [48]. In this regard, we chose the two lipoxygenases, lipoxygenase (1N8Q) and Cytochrome P450 (1OG5). For the first targeted protein, three molecules had a binding affinity that was greater or equal that of the native ligand (in this case, Protocatechuic Acid, with −6.0 kcal/mol); the potent ligand Ascaridole, with −6.8 kcal/mol, establishing three hydrogen bonds (HB) with GLN A:598, VAL A:594, and LYS A:606 (Supplementary Figure S2). In the second protein (CYP450), two molecules were found to be strong inhibitors (*trans*-β-Terpinyl Butanoate, with −6.6 kcal/mol, and α-Terpineol Acetate, with −6.7 kcal/mol, in comparison with Warfarin, with −6.6 kcal/mol (CYP2C9 native ligand)). The binding analysis revealed the formation of a hydrogen bond for both compounds with the amino acid ASN A:217 (Supplementary Figure S3). In such study, the active site of NADPH Oxidase Protein (PDB ID: 2CDU) was found to be surrounded by GLY158, TYR159, ILE160, GLY180, HIS181, TYR188, VAL214, CYS242, and GLY244 (Supplementary Figure S4). None of the docked molecules had free binding energy greater or equal to −8.6 kcal/mol, thus suggesting that DAEOs have no inhibitory potential on NADPH Oxidase Protein. The interactions of the identified components with the fourth protein, Bovine Serum Albumin (PDB: 4JK4), revealed that these phytocompounds act as natural inhibitors of BSA protein (Supplementary Figure S5). Moreover, *m*-Cymene, Carvacrol, and *trans-*β-Terpinyl Butanoate were the three most potent ligands against BSA protein (native ligand: 3,5-Diiodosalicylic Acid; −5.3 kcal/mol), with −7.4, −7.3, and −6.9 kcal/mol, respectively.

The antioxidant potential of the native ligands may be linked to amino acid residues with which they bind. As a result, the analysis of common residues and free binding affinity values around the docked ligands, native inhibitors, and the literature leads to the conclusion that the tested molecules have a good antioxidant activity [49].

DNA gyrase topoisomerase II (PDB: 1KZN), an enzyme of *Escherichia* *coli* bacteria that is composed of two subunits, GyrA (875 amino acids) and GyrB (804 amino acids), was reported to be found in all bacteria regulating the topological state of bacterial DNA [50,51]. This protein was utilized as a binding target protein. Compounds identified in *D. ambrosioides* essential oils demonstrated poor affinities, ranging from −4.6 to −6.2 kcal/mol. On the other hand, the docking of the protein 1KZN with its native ligand Clorobiocin revealed a strong inhibitory potential, where the docking score was −9.6 kcal/mol, establishing two conventional hydrogen bonds with THR165 and ASP73 amino acid residues (Supplementary Figure S6), as reported in Reference [52].

The second protein is Enoyl-Acyl Carrier Protein Reductase (FabI), a critical enzyme in fatty acid synthesis process, namely in type II fatty acid synthase, in which FabI catalyzes the final step required for cycle completion, especially during the elongation phase of fatty acid synthesis [53]. As a result, researchers have increasingly focused their attention on this crucial enzyme for antimicrobial compounds. FabI crystal structures have been found in a variety of bacteria, notably *Escherichia coli* and *Staphylococcus aureus* [54]. From the docking results, we observed that only one molecule (α-Terpineol Acetate) has a binding free energy of −6.2 kcal/mol, equal to that of Triclosan (Supplementary Figure S7), a well-known FabI inhibitor [43].

In the hexosamine biosynthetic pathway (HBP), glucosamine-6-phosphate synthase, known as glmS or (PDB: 2VF5) or glutamine: fructose-6-phosphate amidotransferase (GFAT), catalyzes fructose-6-phosphate (Fru-6-P) and glutamine to glucosamine-6-phosphate (GlcN-6-P). It is a crucial rate-limiting enzyme in HBP that leads to the major end product UDP-N-acetylglucosamine, an important building block of peptidoglycan cell wall bacteria. In *E. coli*, as well as in other bacteria, the product glucosamine-6-phosphate is a feedback inhibitor to glmS gene at post-transcriptional level [55,56,57], considered as the native ligand in this case. All of the tested ligands exhibit a high binding energy compared to the native ligand (−7.2 kcal/mol) (Supplementary Figure S8) [58].

### 2.3. Antioxidant Activity

**Figure 2:**
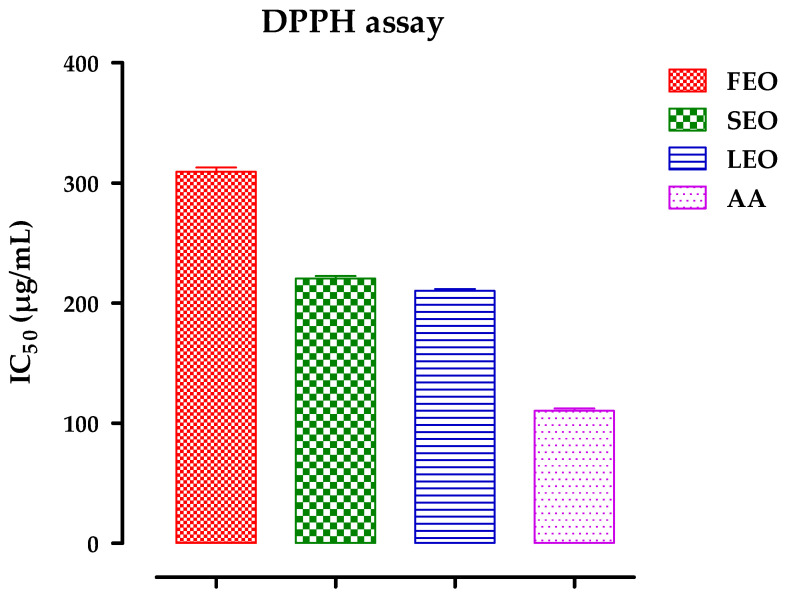
DPPH free radical scavenging activity of DAEOs (FEO, SEO, and LEO), and Ascorbic Acid (AA). The experiment was carried out in a minimum of three repetitions, and the results are reported as mean ± SD.

The antiradical activity of DAEOs was assessed by incubating the flower oils, stem, and leaves with the radical 2,2-diphenyl-1-picrylhydrazyl (DPPH); the radical’s reduction was accompanied by a shift in color from violet to yellow. The findings are recorded in Figure 2, with the inhibitory concentration of 50% of the effect as a function of the concentrations of each oil. These results show that, for the inhibitory concentrations (IC50) of free radicals, which were determined graphically and expressed in µg/mL, *D. ambrosioides* leaf oil showed the greatest trapping capacity of DPPH, with an IC50 of the order of 210.24 ± 2.53 µg/mL, followed by 220.50 ± 3.73 µg/mL for stem oil and 309.45 ± 5.93 for flowers of the same plant. For all tested concentrations, the IC50 of vitamin C (used as a positive control) was lower compared to that of the three essential oils, according to their IC50 values (Figure 2).

**Figure 3:**
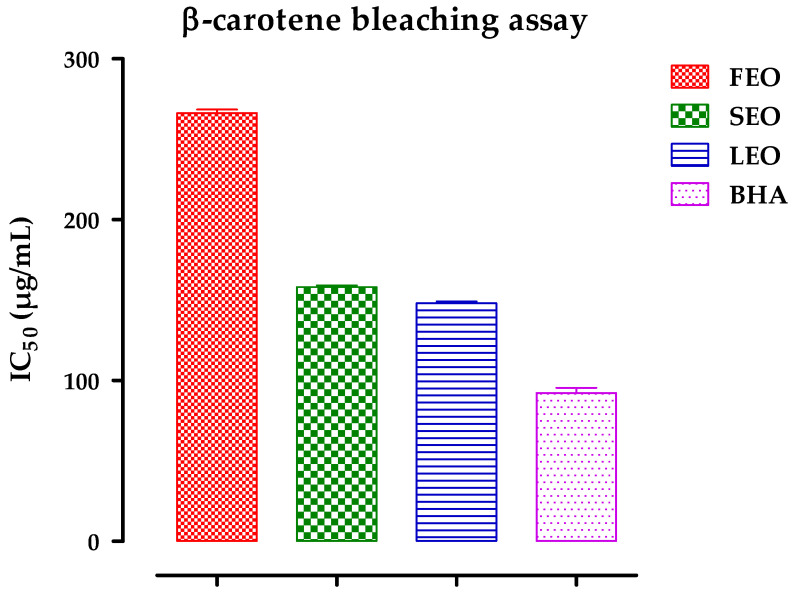
Half inhibition concentration (IC50) of DAEOs (FEO, SEO, and LEO) and butylated hydroxyanisole (BHA). The experiment was carried out in a minimum of triplicate, and the results are reported as mean ± SD.

Figure 3 presents the results of the antioxidant activity of the essential oils of the leaves, flowers, and stems of *D. ambrosioides* by the β-carotene/linolic acid bleaching method. The oil of the leaves and stems showed a greater inhibition activity of bleaching β-carotene 147.99 ± 2.29 and 158.15 ± 1.91 for the leaves and stems respectively than that of flower oil, at 266.25 ± 4.44, but less than that of butylated hydroxyanisole (BHA), which gave a greater inhibition of β-carotene bleaching.

### 2.4. Antibacterial Activity

*D. ambrosioides* essential oils (DAEOs) were tested for antibacterial activity against two strains of Gram-positive pathogenic bacteria (*Enterococcus faecalis* and *Staphylococcus aureus*), one strain of Gram-negative pathogenic bacteria (*Escherichia coli*), and a clinical isolate of *Enterococcus faecalis*, using the agar well diffusion assay method.

Table 3 shows data on the antibacterial properties of DAEOs. *S. aureus* was responsive to all DAEOs tested (IZ = 15 to 20 mm), with the exception of the LEO, which showed moderately sensitive action (IZ = 10 mm). *E. faecalis* (IZ = 8–16 mm) was classified as moderately sensitive to all parts of *D. ambrosioides* in the same table, with the exception of the LEO, which provided fairly sensitive activity (IZ = 9 mm), for *E. coli* (IZ = 16–24 mm). The multi-resistant *E. faecalis* was shown to be sensitive to all DAEOs tested (IZ = 13 to 18 mm). The comparison of inhibitory action by different portions of *D. ambrosioides* was not significant (*p* > 0.05), but the comparison of all strains tested was significant at *p* < 0.05.

Table 4 summarizes the obtained MIC values that were determined on 96-well plates, using the microdilution method. All results indicate that DAEOs exhibit significant inhibitory activity on all strains tested. For the inhibition values for these bacteria, the MIC was 6.0–110.0 μg/mL, and the MBC was 12.0–110.0 μg/mL. It was noted that FEO had the highest antibacterial activity (MIC, 6.0 to 105.0 μg/mL; MBC, 12.0 to 110.0 μg/mL), while LEO had the lowest antibacterial potential (MIC, 105.0 to 110.0 μg/mL; MBC, 110.0 to 110.0 μg/mL). The antibacterial properties of DAEOs tested can be given as FEO < SEO < LEO.

Generally, the antibacterial activity of DAEOs from the three components tested was stronger in Gram-positive bacteria than in Gram-negative bacteria, which might be related to the presence of an inbuilt mechanism of resistance to DAEOs.

Gram-positive bacteria present a peptidoglycan bacterial wall that allows hydrophobic molecules to attain internal environment, while the Gram-negative bacterial wall is composed essentially by lipopolysaccharide which allows small hydrophilic molecules to cross it, due to its abundance of porine proteins, according to Man et al. (2019) [59]. These results are consistent with those reported by Alitonou et al. (2012), who evaluated *D. ambrosioides* essential oil against one Gram-positive bacteria, *S. aureus*, and one Gram-negative bacteria, *E. coli* [60].

Baumgart (2014) conducted microbiological studies on *D. ambrosioides* leaves essential oil against conventional bacterial strains, such as Gram-positive *S. aureus* ATCC 6538P and Gram-negative *E. coli* ATCC 11775, among other bacteria; no antibacterial activity was discovered; an MIC of 1024 g/mL was considered inactive against these bacteria, and this contradicts the findings of this study [61]. *E. faecalis* was found to be the most resistant to DAEOs tested. The structure of the bacterial cell wall is known to make it sensitive to the effect of essential oils. A correlation between the antibacterial activity of DAEOs in this work and their p suggests that the activity of the oils might be attributed to the presence of the main components of the oil (Carene, Carvacrol, Cymene, Thymol, and Camphor) in all tested DAEOs. The results obtained in this work demonstrate the high antibacterial activity of FEO and SEO in comparison with Tetracycline (bacteriostatic antibiotic of the cyclin class), Ampicillin (a beta-lactam antibiotic with a broad spectrum of action against Gram-positive and Gram-negative bacteria), and Azithromycin (an effective antibiotic used to treat a variety of bacterial infections) (20.11, 18.08, and 20.97 µg/mL, respectively) tested on *E. coli* ATCC 10536, as well as in *S. aureus* ATCC 6538, by Abd-ElGawad et al. (2022) [62]. The clinical isolate of *E. faecalis* was found to be sensitive to SEO at a concentration of 18 µg/mL. SEO displayed a potent antibacterial effect compared to Ciprofloxacin (a bactericidal agent that inhibits cell multiplication by neutralizing bacterial replication enzymes), which was used as a positive control against *E. faecalis* ATCC 9854 (24.66 µg/mL) in Mahmoudzadeh et al. (2016) work [63].

### 2.5. ADMET Analysis

A medicine’s restricted absorption, distribution, metabolism, excretion, and toxicity (ADMET) characteristics may risk its effectiveness. Furthermore, it is regarded that the most significant drawback for drug development in clinical research is its pharmacokinetic characteristics, which make it extremely costly. As a consequence, in silico approaches were used to assess ADMET features in order to predict the chance of *D. ambrosioides* essential oils being a drug development candidate.

As part of this study, several properties were considered, such as physicochemical properties, absorption, distribution, metabolism, and toxicity. The compounds were evaluated under the parameters shown above (Table 5). In order to satisfy Lipinski’s rule of five, some physicochemical properties are required (<5 H-bond donors, <10 H-bond acceptors, N or O ≤ 10, MW < 500 Da, and MLOGP ≤ 4.15) [64]. Surprisingly, all the phytoconstituents (1–15) above fulfill Lipinski’s rule of five. However, compounds 1 and 2 display one violation by exceeding the lipophilicity threshold (MLOGP > 4.15). According to Martin (2005), any compound adhering to Lipinski’s rule of five has 0.55 set as a bioavailability score [65]. Consequently, the bioavailability score is 0.55.

According to the logS scale, compounds with water solubility (given as logS (log mol/L)) ranging from –4 to 0 have good solubility [66]. Accordingly, the phytochemicals in this case are considered to be good water-soluble compounds.

All of the phytochemical compounds above show high Caco2 permeability (given as log P in 10^−6^ cm/s); thus, they have a high percentage of human intestinal absorption. The absorption results showed that none of the compounds came out to be a P-glycoprotein substrate nor a P-glycoprotein I and II inhibitor. VDss (expressed as Log L/Kg) stands for the volume of distribution at steady-state in humans. Reportedly, the compounds show a good distribution in the plasma. Compounds 4 and 9 display the best penetration through the blood–brain barrier with the highest logBB, while the Central Nervous System (CNS) permeability is considered reasonable for all of them. Regarding metabolism, it is essential to define metabolic interactions between the compounds and the cytochrome P450, indicating whether the molecule is an inhibitor or a substrate to the main two isoenzymes, CYP2D6 and CYP3A4, which have an significant function in drugs’ metabolism [67]. None of the identified components above is a substrate or an inhibitor of CYP2D6 and CYP3A4. The constituents (1–15) came out to be non-substrates for Renal OCT2 (Organic Cation Transporter 2), whereas the best total clearance (mL/min/kg) was observed in α-Terpineol Acetate (11) and Carvenone Oxide (12).

The AMES toxicity indicates mutagenic and carcinogenic compounds. The 1-(4-Bromobutyl)-2-Piperidinone is the only compound that is AMES positive, while the others are AMES negative. Except for the compounds 7 and 13, none of the molecules in the table appears to be distorting human liver functions. Ventricular arrhythmia can be caused through the inhibition of hERG by blocking potassium channels [68]; nevertheless, none of the compounds inhibits this gene. All the phytochemical compounds above, besides 1 and 4, may induce allergic contact dermatitis.

**Figure 4:**
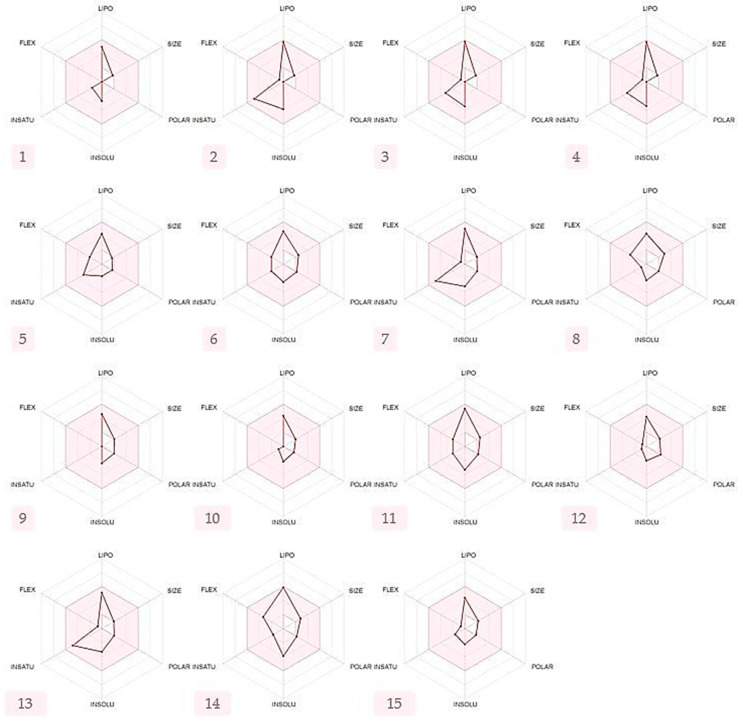
Phytoconstituents bioavailability radars considering six physicochemical properties (lipophilicity, size, polarity, solubility, flexibility, and saturation) ideal for oral bioavailability. Note: (1) (+)-4-Carene, (2) *m*-Cymene, (3) D-Limonene, (4) γ-Terpinene, (5) 5-Methyl-3-Hepten-2-One, (6) α-Cyclogeraniol Acetate, (7) Thymol, (8) 1-(4-Bromobutyl)-2-Piperidinone, (9) *(1R,2R,3R,5S)*-(-)-Isopinocampheol, (10) Camphor, (11) α-Terpineol Acetate, (12) Carvenone Oxide, (13) Carvacrol, (14) *trans*-β-Terpinyl Butanoate, and (15) Ascaridole.

Figure 4 shows the bioavailability radars of the identified compounds; the pink area represents the oral bioavailability space where the molecule’s graph has to completely fit in order to be asserted drug-like. In the present investigation, all the phytocompounds adhere to the suitable space for oral bioavailability, as shown above.

**Figure 5:**
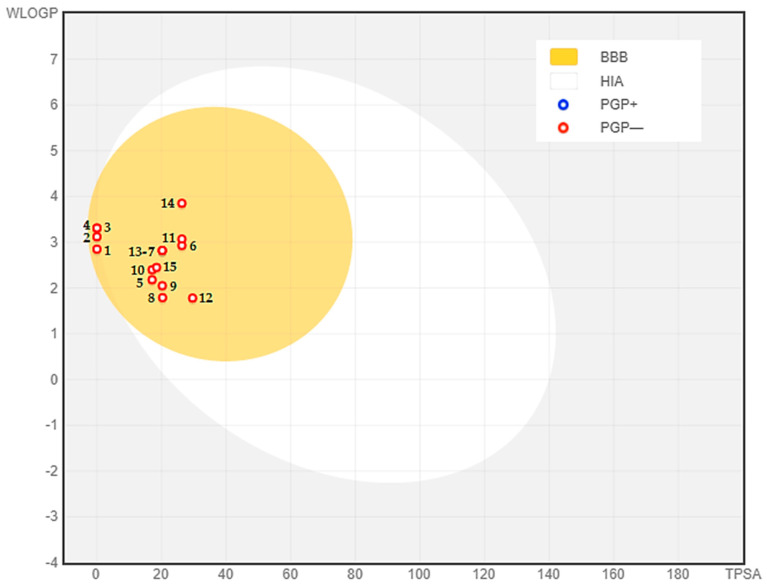
BOILED-Egg model of DAEOs phytocompounds, using Swiss ADME predictor. Note: (1) (+)-4-Carene, (2) *m*-Cymene, (3) D-Limonene, (4) γ-Terpinene, (5) 5-Methyl-3-Hepten-2-One, (6) α-Cyclogeraniol Acetate, (7) Thymol, (8) 1-(4-Bromobutyl)-2-Piperidinone, (9) *(1R,2R,3R,5S)*-(-)-Isopinocampheol, (10) Camphor, (11) α-Terpineol Acetate, (12) Carvenone Oxide, (13) Carvacrol, (14) *trans*-β-Terpinyl Butanoate, and(15) Ascaridole.

The BOILED-Egg model provides a first glance to assess intestinal absorption (IA) and blood–brain barrier permeability (BBB) based on lipophilicity (WLOGP) and polarity (TPSA) [69]. The white area represents molecules with strong intestinal absorption, whereas the molecules inside the yolk (yellow area) indicate high BBB permeability (Figure 5). The color of the dots shows whether a molecule is a P-glycoprotein substrate (blue) or a P-glycoprotein non-substrate (red). To this instance, the phytocompounds are identified as well-absorbed and excellent penetrant to the blood–brain barrier and found to be non-substrate to P-glycoprotein.

## 3. Materials and Methods

### 3.1. Plant Material, Extraction, and Yielding of D. ambrosioides Essential Oils

The different parts (leaves, stems, and flowers) of *Dysphania ambrosioides* were collected near “Guercif” (Eastern Morocco) in February 2021. The botanical identification was carried out at the Department of Biology of the Faculty of Sciences, University Mohammed the first (Oujda, Morocco), where a voucher specimen was deposited under the collection number HUMPOM44.

Different portions of *D. ambrosioides* were isolated and dried naturally in a covered room until steady weight (about 6 days). Before hydrodistillation, the thoroughly dried samples were crushed into a fine powder. A Clevenger type equipment was used to hydrodistill 100 g of plant material in 300 mL of water until the essential oil content was steady (2 to 3 h).

Following extraction, anhydrous sodium sulfate was employed to eliminate any remaining water. The following formula is used to compute the extraction yields:

Yield (%) = (M_extract_/M_sample_) × 100

where M_extract_ is the mass of oil in grams, and M_sample_ is the mass of the sample (plant) in grams.

Lastly, until analysis, the essential oil was kept in an airtight glass container in a refrigerator at 4 °C.

### 3.2. GC–MS Analysis

A Shimadzu GC system (Kyoto, Japan) with a BPX25 capillary column with a 95 percent dimethylpolysiloxane diphenyl phase (30 m 0.25 mm ID 0.25 m film thickness) and an MS QP2010 was used for separation and identification. As a carrier gas, pure helium (99.99 percent) was employed at a constant flow rate of 3 mL/min. The mass range studied was 40 to 300 *m*/*z*, and 1 L of each produced oil was fed into the chamber and diluted with an appropriate solvent. A total of 1 μL of each prepared oil diluted with an appropriate solvent was injected in the fractionation mode (fractionation ratio 90:1). Samples were evaluated three times. Finally, the compounds were recognized by comparing their retention times to verified standards, and their mass spectrum fragmentation models to those found in databases or on NIST compounds. Data were collected and processed by using Laboratory Solutions (v2.5).

### 3.3. Molecular Docking

The molecular docking analysis was performed as described in Reference [70]. Protein structures of four antioxidant proteins (namely, Lipoxygenase (PDB: 1N8Q), CYP2C9 (PDB: 1OG5), NADPH Oxidase (PDB: 2CDU), and Bovine Serum Albumin (PDB: 4JK4)) and three antimicrobial proteins (DNA gyrase topoisomerase II from *E. coli* (PDB: 1KZN), Enoyl-Acyl Carrier Protein Reductase from *S. aureus* (PDB: 3GNS), and Glucosamine-6-Phosphate (PDB: 2VF5)) were retrieved from RCSB Protein Data Bank (PDB) in a crystallographic 3D structure and adopted as docking targets, using Autodock Tools (version 1.5.6). The protein structures were stripped of H_2_O molecules, metal atoms, co-crystalized ligands, and other non-covalently bound substances. Following the addition of Kollman charges, polar hydrogens and the merge of nonpolar hydrogens, the target file was saved as an appropriate pdbqt format. Ligands identified in *D. ambrosioides* essential oils were constructed as follows: sdf (3D conformer) file was downloaded from PubChem (https://pubchem.ncbi.nlm.nih.gov/) (accessed on 5 March 2022) and then converted to a pdb file by using PyMol. The ligand final pdbqt file was obtained by using Autodock Tools (version 1.5.6). Rigid molecular docking was executed with Autodock Vina’s embedded scoring function [71]. The grid box representing the docking search space was resized to best match the active binding site. Table 6 shows the grid box coordinates. The docked ligand complexes’ data were given as ΔG binding energy values (kcal/mol). Discovery Studio 4.1 (Dassault Systems Biovia, San Diego, CA, USA) was used to examine protein–ligand binding interactions and in the construction of 2D schemes of molecular interactions.

### 3.4. Antioxidant Assays

#### 3.4.1. The 2,2-diphenyl-1-picrylhydrazyl (DPPH) Assay

The determination of the free radical trapping capacity (DPPH) of different EOs tested was carried out according to References [74,75,76], but with some modifications. After the preparation of the DPPH solution by solubilizing 2 mg of DPPH in 100 mL of methanol, another preparation of a range of final concentrations of the tested extracts (25, 50, 100, 150, 200, 250, 300, 350, and 400 µg/mL) was carried out. Subsequently, 2.5 mL of the DPPH solution was added to each volume of the final extract concentration to make a final volume of 3 mL. The same experimental protocol was followed for the standard test, Ascorbic Acid. The samples were then incubated in the dark for 30 min at room temperature. A spectrophotometer was used to measure the absorbance at 517 nm compared to a blank measurement containing methanol. For each concentration of the anti-radical extracts and positive control tests, the measurements were conducted in triplicate. The results were denoted as a percentage of anti-radical activity, and the inhibitory IC50 concentration was automatically calculated by the statistical software.The antiradical activity was estimated according to the equation below:

$$\mathrm{Radical}\;\mathrm{Scavenging}\;\mathrm{Activity}\;\left(\%\right)=\left[\left(\frac{A\;\mathrm{control}-A\;\mathrm{sample}}{A\;\mathrm{control}}\right)\right]\times 100$$

#### 3.4.2. β-Carotene/Linoleic Acid Bleaching Assay

The bleaching test for β-carotene was performed by using the method described by Reference [77], but with some modifications. The preparation of β-carotene solution was prepared by dissolving 2 mg of β-carotene powder in 10 mL of chloroform; subsequently, 20 mg of linoleic acid and 200 mg of the Tween-80 emulsifier were added to the β-carotene solution. For 5 min, a rotary evaporator at 40 °C and 80 rpm was used to eliminate chloroform. β-carotene solution was reconstituted by adding 100 mL of distilled water, while maintaining vigorous agitation. A volume of 2 mL of this emulsion was added to the various extract quantities to yield a final amount of 2.5 mL. All tubes were incubated in a 50 °C water bath with constant agitation for 2 h. Absorbance was measured at 470 nm immediately after the addition of emulsion (t_0_) and after two hours of incubation (t_1_), both against a white reading containing all of the components of the prior emulsion but no β-carotene. As a control, butyl hydroxyanisole (BHA) was utilized. All measurements were carried out in triplicate. The residual color percentage was computed by using the following formula:

$$\mathrm{Residual}\;\mathrm{Color}\;\left(\%\right)=\left[\left(\frac{\mathrm{initial}\;\mathrm{OD}-\mathrm{sample}\;\mathrm{OD}}{\mathrm{initial}\;\mathrm{OD}}\right)\right]\times 100$$

### 3.5. Bacterial Strains, Growth Media, and Chemicals

#### 3.5.1. Bacterial Strains

The antibacterial activity of different parts of *D. ambrosioides* of the essential oil were tested on four bacterial strains, including Gram-positive bacteria, namely *Staphylococcus aureus* ATCC29213, *Enterococcus faecalis* ATCC29212; a Gram-negative bacterium, *E. coli* ATCC25922; and *E. faecalis* clinically isolated at the microbiology laboratory of the Mohammed VI University Hospital Centre in Oujda (Morocco).

#### 3.5.2. Growth Medium

The bacterial strains were cultured by seeding an isolated colony from the nutrient agar boxes in 50 mL of TSB medium. The broth was then incubated for 24 h at 37 and 30 °C for Listeria strains, until the stationary growth phase was reached. The antibacterial activity of essential oils was evaluated in vitro via two techniques, as detailed below.

The agar diffusion method was used as a pre-test for a qualitative determination of the antibacterial potential of the DAEOs. After obtaining the bacterial strains, 0.1 mL (107 CFU/mL) of the bacterial suspension of each strain of interest was inoculated on the surface of a box of Mueller–Hinton Agar (MHA), using a display stand to have a homogeneous distribution of growth throughout the box. Sterile filter paper discs (Wattman N°1, 6 mm diameter) were individually impregnated with 20 μL of each essential oil dissolved in 2% DMSO and then placed with a sterile clamp on the surface of the MHA Petri dishes previously seeded with the microorganisms. Petri dishes were kept at 4 °C for 2 h to allow the diffusion of essential oils. They were then incubated at 37 °C for 18 to 24 h. The diameters of the resulting inhibition zones were measured in mm. The tests were performed in triplicate. The results are expressed as an average of three determinations (±) standard deviation (SD).

#### 3.5.3. Determination of Minimum Inhibitory Concentrations (MIC)

In this work, the micro-dilution technique using Resazurin as an indicator of bacterial growth [78] was used to determine MIC values. The use of Resazurin as a visual indicator for determining the MIC values of essential oils is a novel method of micro-dilution. It is an oxy-reduction indicator used for the evaluation of cell growth. It is a non-fluorescent violet/blue dye that becomes pink and fluorescent when reduced to resorufin by oxidoreductase enzymes in viable cells [79].

Essential oils stock solutions were prepared by dissolving 2 g of each oil in 100 mL of DMSO; the final concentration of each stock solution was 2%. A series of dilutions ranging from 2 to 120 μg/mL were prepared in Mueller–Hinton Broth (MHB). After stirring, 180 μL of each concentration was added to the wells of a 96-well microtitration plate. The prepared microbial suspensions were diluted to a concentration of 107 CFU/mL, and 20 μL was added to each well (final concentration of 106 CFU/mL). All tests were conducted in triplicate. For each analysis, the following controls were prepared:the positive controlincluded MHB and DMSO with the microorganism under study, and thenegative controlincluded MHB medium, with the addition of essential oils, but without bacterial suspension.

The microtitration plates were then incubated at 37 °C for 18 to 24 h at the optimal temperature for each germ. Each well received 5 L of Resazurin at 0.01% (*w*/*v*) for the MIC disclosure. The MIC is defined as the least essential oil concentration that does not modify the staining of Resazurin and corresponds to the lack of bacterial growth.

#### 3.5.4. Determination of Minimum Bactericidal Concentrations (MBC)

MBC is defined as the minimum bactericidal concentration of oil that is capable of killing 99% of the inoculum. From the wells where there was no change in coloration and, therefore, no growth, aliquots of 10 μL from each well were transferred and sown on the Mueller–Hinton agar (MHA) and then incubated for 18 h at the appropriate temperatures for each germ. MBC was the smallest concentration where there was no subculture.

#### 3.5.5. Chemicals

Reagents and solvents were purchased from Sigma-Aldrich Chemicals (Inc., St. Louis, MO, USA): Hexane, Dimethyl Sulfoxide (DMSO), Sodium Sulfate, and Resazurin.

### 3.6. ADMET Analysis

The pharmacokinetic properties (absorption, distribution, metabolism, and excretion) and toxicity of the compounds from *D. ambrosioides* essential oils through GC–MS were predicted by using the SwissADME (http://www.swissadme.ch/) (accessed on 10 March 2022) and pkCSM (http://biosig.unimelb.edu.au/pkcsm/prediction) (accessed on 10 March 2022) online tools [80].

## 4. Conclusions

According to the current study, the essential oils isolated from *D. ambrosioides’* portions are high in monoterpenes compounds, particularly 4-(+)-Carene, α-Cyclogeraniol Acetate, *m*-Cymene, α-Terpineol Acetate, and *trans*-β-Terpinyl Butanoate, among other compounds. *D. ambrosioides* essential oils have a broad-spectrum antibacterial effect against two Gram-positive bacteria (*E. faecalis* and *S. aureus*), a clinical isolate of *E. faecalis*, and one Gram-negative pathogenic bacteria (*E. coli*), along with a mild-to-potent antioxidant potential, which was unveiled by DPPH, as well as β-carotene/linoleic acid bleaching assays. As a result, the DAEOs under study could be used in a variety of food and therapeuticalapplications as an innovative source of natural preservatives and antioxidants.

## Figures and Tables

**Table 1:** Phytoconstituents identified from three essential oils (SEO, LEO, and FEO) of *D. ambrosioides* by GC–MS analysis.

Compound Number	Compound Name	Formula	Mol. Wt.	RT (min)	Peak Area (%)
Stem Essential Oil (SEO)					
1	(+)-4-Carene	C_10_H_16_	136.23	6.467	50.5
2	*m*-Cymene	C_10_H_14_	134.22	6.592	3.13
3	D-Limonene	C_10_H_16_	136.23	6.667	0.97
4	γ-Terpinene	C_10_H_16_	136.23	7.158	0.68
5	3-Hepten-2-one, 5-Methyl	C_8_H_14_O	126.20	8.2330	2.06
6	α-Cyclogeraniol Acetate	C_12_H_20_O_2_	196.29	10.108	22.64
7	Thymol	C_10_H_14_O	150.22	10.800	7.16
8	1-(4-Bromobutyl)-2-Piperidinone	C_9_H_16_BrNO	234.13	10.958	3.98
9	*(1R,2R,3R,5S)*-(-)-Isopinocampheol	C_10_H_18_O	154.25	11.108	8.87
Leaves Essential Oil (LEO)					
10	α-Terpinene	C_10_H_16_	136.23	6.367	5.67
11	(+)-4-Carene	C_10_H_16_	136.23	6.467	46.2
12	*m*-Cymene	C_10_H_14_	134.22	6.592	20.74
13	D-Limonene	C_10_H_16_	136.23	6.667	1.38
14	γ-Terpinene	C_10_H_16_	136.23	7.158	0.69
15	Camphor	C_10_H_16_O	152.23	8.642	0.20
16	α-Terpineol Acetate	C_12_H_20_O_2_	196.29	10.108	14.5
17	Carvenone Oxide	C_10_H_16_O_2_	168.23	10.367	0.54
18	Thymol	C_10_H_14_O	150.22	10.800	3.76
19	Carvacrol	C_10_H_14_O	150.22	10.958	1.73
20	*(1R,2R,3R,5S)*-(-)-Isopinocampheol	C_10_H_18_O	154.25	11.108	4.42
Flowers Essential Oil (FEO)					
21	(+)-4-Carene	C_10_H_16_	136.23	6.467	28.05
22	*m*-Cymene	C_10_H_14_	134.22	6.592	8.15
23	D-Limonene	C_10_H_16_	136.23	6.658	1.12
24	*trans*-β-Terpinyl Butanoate	C_14_H_24_O_2_	224.34	10.117	31.13
25	Ascaridole	C_10_H_16_O_2_	168.23	10.367	1.16
26	Thymol	C_10_H_14_O	150.22	10.800	7.79
27	Carvacrol	C_10_H_14_O	150.22	10.950	4.53
28	*(1R,2R,3R,5S)*-(-)-Isopinocampheol	C_10_H_18_O	154.25	11.117	18.06

**Table 2:** Heatmapofthedockingscores (bindingfreeenergy values are expressed in kcal/mol) of *D. ambrosioides* essential oils components: 1N8Q, Lipoxygenase; 1OG5, CYP2C9; 2CDU, NADPH Oxidase; 4JK4, Bovine Serum Albumin; 1KZN, DNA Gyrase Topoisomerase II; 3GNS, Enoyl-Acyl Carrier Protein Reductase; 2VF5, Glucosamine-6-Phosphate Synthase.

Ligand	Antioxidant Proteins PDB IDs				Antibacterial Proteins PDB IDs		
	1N8Q	1OG5	2CDU	4JK4	1KZN	3GNS	2VF5
	Free Binding Energy ∆G (kcal/mol) ^1^						
Native Ligand	−6.0	−6.6	−8.6	−5.3	−9.6	−6.0	−7.2
(+)-4-Carene	−6.1	−5.8	−5.8	−6.2	−5.0	−5.0	−5.1
*m*-Cymene	−5.5	−5.9	−5.8	−7.4	−4.8	−4.9	−5.1
D-Limonene	−6.0	−6.3	−5.6	−6.3	−5.8	−4.7	−5.0
γ-Terpinene	−5.1	−6.1	−5.6	−6.4	−5.8	−4.7	−5.0
3-Hepten-2-one, 5-Methyl	−4.5	−4.8	−5.0	−5.9	−4.8	−4.3	−4.1
α-Cyclogeraniol Acetate	−5.8	−6.2	−5.6	−6.1	−5.8	−5.5	−6.2
Thymol	−5.3	−6.0	−5.5	−6.2	−6.2	−5.1	−5.2
1-(4-Bromobutyl)-2-Piperidinone	−4.8	−5.4	−5.1	−5.4	−4.7	−4.0	−4.7
*(1R,2R,3R,5S)*-(-)-Isopinocampheol	−5.2	−5.7	−5.9	−6.1	−4.7	−4.6	−5.4
Camphor	−5.5	−5.9	−5.6	−5.7	−4.6	−5.2	−5.9
α-Terpineol Acetate	−5.9	−6.7	−6.4	−6.5	−6.1	−6.0	−5.9
Carvenone Oxide	−5.9	−5.7	−5.9	−6.1	−4.7	−5.3	−5.7
Carvacrol	−6.2	−6.2	−6.0	−7.3	−6.0	−5.4	−5.3
*trans*-β-Terpinyl Butanoate	−6.1	−6.6	−6.1	−6.9	−6.4	−5.2	−6.2
Ascaridole	−6.8	−5.8	−6.1	−6.5	−5.2	−5.4	−5.6

^1^ For each column, the color scale ranges from red (referring to the native ligand ∆G), through yellow (mid-point), to green (native ligand ∆G + 5 kcal/mol).

**Table 3:** Determination of inhibition zones of *D. ambrosioides* essential oils.

Bacteria	Inhibition Zones (IZ) of *D. ambrosioides* Essential Oils		
	SEO IZ (mm)	LEO IZ (mm)	FEO IZ (mm)
*E. coli*	16 ± 0.23	9 ± 0.20	24 ± 0.10
*S. aureus*	15 ± 0.11	10 ± 0.31	20 ± 0.00
*E. faecalis* ^1^	8.0 ± 0.11	9 ± 0.31	16 ± 0.00
*E. faecalis* ^2^	18 ± 0.40	13 ± 0.21	14 ± 0.20

^1^*Enterococcus faecalis* ATCC 29212; ^2^ Enterococcusfaecalis clinical isolate.

**Table 4:** Determination of MIC and MBC of *D. ambrosioides* essential oils.

Bacteria	Essential Oils of *D. ambrosioides*					
	SEO		LEO		FEO	
	MIC (µg/mL)	MBC (µg/mL)	MIC (µg/mL)	MBC (µg/mL)	MIC (µg/mL)	MBC (µg/mL)
*E. coli*	18	18	105	110	6	12
*S. aureus*	18	18	110	110	12	18
*E. faecalis* ^1^	≥110	≥110	≥110	≥110	105	110
*E. faecalis* ^2^	18	18	105	≥110	105	105

^1^*Enterococcus faecalis* ATCC 29212; ^2^ Enterococcus faecalisclinical isolate.

**Table 5:** ADMETpropertiesof theidentifiedphytochemicalsof DAEOs:(1) (+)-4-Carene, (2) *m*-Cymene, (3) D-Limonene, (4) γ-Terpinene, (5) 5-Methyl-3-Hepten-2-One, (6) α-Cyclogeraniol Acetate, (7) Thymol, (8) 1-(4-Bromobutyl)-2-Piperidinone, (9) *(1R,2R,3R,5S)*-(-)-Isopinocampheol, (10) Camphor, (11) α-Terpineol Acetate, (12) Carvenone Oxide, (13) Carvacrol, (14) *trans*-β-Terpinyl Butanoate, and (15) Ascaridole.

Compound N.		1	2	3	4	5	6	7	8	9	10	11	12	13	14	15
Drug-Likeness	Lipinski’s rule of five	Yes	Yes	Yes	Yes	Yes	Yes	Yes	Yes	Yes	Yes	Yes	Yes	Yes	Yes	Yes
	Bioavailability Score (%)	0.55	0.55	0.55	0.55	0.55	0.55	0.55	0.55	0.55	0.55	0.55	0.55	0.55	0.55	0.55
Absorption	Water Solubility	−2.74	−3.89	−3.50	−3.45	−1.73	−2.57	−3.19	−2.35	−2.40	−2.16	−3.35	−2.04	−3.31	−3.92	−2.23
	Caco2 Permeability	1.39	1.52	1.40	1.41	1.51	1.63	1.60	1.35	1.47	1.49	1.62	1.51	1.60	1.65	1.61
	Intestinal Absorption (Human) (%)	96.3	93.6	95.8	96.2	96.3	96.6	90.8	93.1	94.2	95.9	96.2	98.2	90.8	95.3	96.3
	Skin Permeability	−4.82	−3.92	−3.89	−3.94	−5.60	−5.57	−4.87	−6.24	−5.43	−5.67	−4.69	−5.95	−4.74	−4.27	−5.73
	P-glycoprotein Substrate	No	No	No	No	No	No	No	No	No	No	No	No	No	No	No
	P-glycoprotein I Inhibitor	No	No	No	No	No	No	No	No	No	No	No	No	No	No	No
	P-glycoprotein II Inhibitor	No	No	No	No	No	No	No	No	No	No	No	No	No	No	No
Distribution	VDss (human)	0.51	0.72	0.39	0.41	0.06	0.15	0.51	0.10	0.47	0.33	0.13	0.20	0.51	0.26	0.35
	BBB permeability	0.76	0.47	0.73	0.75	0.49	0.51	0.40	0.58	0.75	0.61	0.42	0.55	0.40	0.53	0.63
	CNS permeability	−2.25	−1.39	−2.37	−2.04	−2.17	−2.67	−1.66	−2.61	−2.45	−2.15	−2.84	−2.51	−1.66	−2.72	−2.74
Metabolism	CYP2D6 Substrate	No	No	No	No	No	No	No	No	No	No	No	No	No	No	No
	CYP3A4 Substrate	No	No	No	No	No	No	No	No	No	No	No	No	No	No	No
	CYP2D6 Inhibitor	No	No	No	No	No	No	No	No	No	No	No	No	No	No	No
	CYP3A4 Inhibitor	No	No	No	No	No	No	No	No	No	No	No	No	No	No	No
Excretion	Total Clearance	0.02	0.24	0.21	0.21	0.33	0.37	0.21	0.28	0.01	0.10	1.24	1.14	0.20	1.31	1.33
	Renal OCT2 Substrate	No	No	No	No	No	No	No	No	No	No	No	No	No	No	No
Toxicity	AMES Toxicity	No	No	No	No	No	No	No	Yes	No	No	No	Yes	No	No	No
	Hepatotoxicity	No	No	No	No	No	No	Yes	No	No	No	No	No	Yes	No	No
	hERG I Inhibitor	No	No	No	No	No	No	No	No	No	No	No	No	No	No	No
	Skin Sensitization	No	Yes	Yes	No	Yes	Yes	Yes	Yes	Yes	Yes	Yes	Yes	Yes	Yes	Yes

**Table 6:** Molecular modeling proteins and grid-box parameters.

Proteins	PDB ID	Grid Box Size	Grid Box Center	Native Ligand	Reference
Lipoxygenase	**1N8Q**	size_x = 40	center_x = 22.455	Protocatechuic Acid	[71]
		size_y = 40	center_y = 1.2930		
		size_z = 40	center_z = 20.362		
CYP2C9	**1OG5**	size_x = 12.387	center_x = −19.823	Warfarin	[71]
		size_y = 11.653	center_y = 86.686		
		size_z = 11.654	center_z = 38.275		
NADPH Oxidase	**2CDU**	size_x = 14.007	center_x = 18.997	Adenosine-5′-Diphosphate	[71,72]
		size_y = 14.997	center_y = −5.777		
		size_z = 18.795	center_z = −1.808		
Bovine Serum Albumin (BSA)	**4JK4**	size_x = 40	center_x = 95.873	3,5-Diiodosalicylic Acid	[73]
		size_y = 40	center_y = 16.048		
		size_z = 40	center_z = 13.494		
DNA Gyrase Topoisomerase II (*E. coli*)	**1KZN**	size_x = 40	center_x = 19.528	Clorobiocin	[40]
		size_y = 40	center_y = 19.500		
		size_z = 40	center_z = 43.031		
Enoyl-Acyl Carrier Protein Reductase (*S. aureus*)	**3GNS**	size_x = 40	center_x = −14.280	Triclosan	[42,43]
		size_y = 40	center_y = 0.56200		
		size_z = 40	center_z = −21.462		
Glucosamine-6-Phosphate Synthase	**2VF5**	size_x = 70	center_x = 30.590	Glucosamine-6-Phosphate	[41]
		size_y = 64	center_y = 15.822		
		size_z = 56	center_z = 3.4970		

## Data Availability

All the data supporting the findings of this study are included in this article.

## Supplementary Materials

The following supporting information can be downloaded at https://www.mdpi.com/article/10.3390/antibiotics11040482/s1. Figure S1: Gas chromatograms of *Dysphania ambrosioides* essential oils:(a) stem essential oil, (b) leaves essential oil, and (c) flowers essential oil. Figure S2: Two-dimensional binding interactions of compounds D-Limonene (A), 4-(+)-Carene (B), Carvacrol (C), trans-β-Terpinyl Butanoate (D), Ascaridole (E), and Protocatechuic Acid (native ligand) (F) against Lipoxygenase protein (PDB ID: 1N8Q). Figure S3: Two-dimensional binding interactions of compounds trans-β-Terpinyl Butanoate (A), α-Terpineol Acetate (B), and Warfarin (native ligand) (C) against CYP2C9 protein (PDB ID: 1OG5). Figure S4: Two-dimensional binding interactions of compounds α-Terpineol Acetate (A) and Adenosine-5′-Diphosphate (native ligand) (B) against NADPH Oxidase Protein (PDB ID: 2CDU). Figure S5: Two-dimensional binding interactions of compounds *m*-Cymene (A), Carvacrol (B), *trans*-β-Terpinyl Butanoate (C), and Adenosine-5′-Diphosphate (native ligand) (D) against Bovine Serum Albumin (BSA) protein (PDB ID: 4JK4). Figure S6: Two-dimensional binding interactions of compounds trans-β-Terpinyl Butanoate (A) and Clorobiocin (native ligand) (B) against DNA Gyrase Topoisomerase II (*E. coli*) protein (PDB ID: 1KZN). Figure S7: Two-dimensional binding interactions of compound α-Terpineol Acetate (A) and Triclosan (native ligand) (B) against S. aureus Enoyl-Acyl Carrier Protein Reductase (FabI) protein (PDB ID: 3GNS). Figure S8: Two-dimensional binding interactions of compound α-Cyclogeraniol Acetate (A) and Glucosamine-6-Phosphate (native ligand) (B) against Glucosamine-6-Phosphate Synthase Protein (PDB ID: 2VF5).
