# Supplementary material for: Antibacterial and Antioxidant Activity of Dysphania ambrosioides (L.) Mosyakin and Clemants Essential Oils: Experimental and Computational Approaches
Source: Antibiotics (Basel). 2022 Apr 5;11(4):482. doi: 10.3390/antibiotics11040482 (PMC9031865; doi:10.3390/antibiotics11040482)
Supplement: Supplementary file 1 [file antibiotics-11-00482-s001.zip › antibiotics-1668866-supplementary.pdf]

antibiotics

MDPI

Supplementary file

Antimicrobial and Antioxidant Activity of *Dysphania ambrosioides* (L.) Mosyakin & Clemants Essential Oils: Experimental and Computational Approaches

Fahd Kandsi [1], Amine Elbouzidi [2], Fatima Zahra Lafdil [1], Nada Meskali [2], Ali Azghar [3], Mohamed Addi [2,*], Christophe Hano [4,5,*], Adil Malab [3], and Nadia Gseyra [1]

[1] Laboratory of Bioresources, Biotechnology, Ethnopharmacology and Health, Faculty of Sciences, Mohammed First University, B.P. 717, Oujda 60000, Morocco; Kandsifahd1994@gmail.com (F.K.); Lafdil.fatimazahra@ump.ac.ma (F.Z.L.); a.azghar@ump.ac.ma (A.A.); ngseyra@hotmail.com (N.G.)
[2] Laboratoire d'Amélioration des Productions Agricoles, Biotechnologie et Environnement (LAPABE), Faculté des Sciences, Université Mohammed Premier, Oujda 60000, Morocco; amine.elbouzidi@ump.ac.ma (A.E.); nada.meskali@gmail.com (N.M.)
[3] Laboratoire de Microbiologie, Centre Hospitalier Universitaire (CHU), Oujda, Morocco.
[4] Laboratoire de Biologie des Ligneux et des Grandes Cultures, INRAE USC1328, University of Orleans, CEDEX 2, 45067 Orléans, France
[5] Le StudiumInstitue for Advanced Studies, 1 Rue Dupanloup, 45000 Orléans, France

* Correspondence: m.addi@ump.ac.ma (M.A.) ; Christophe.hano@univorleans.fr (C.H.)

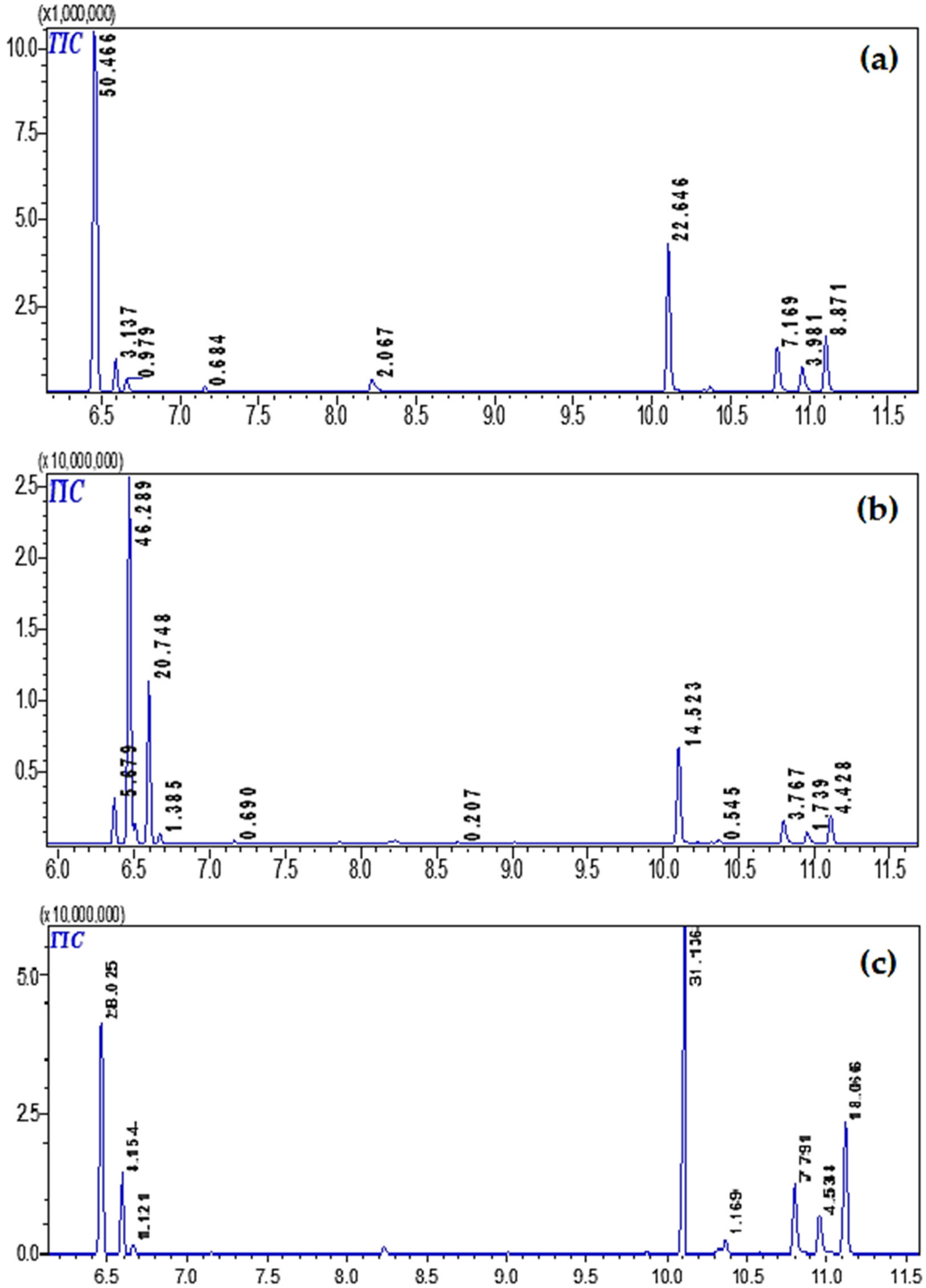

Figure S1. Gas chromatograms of *Dysphania ambrosioides* essential oils; **(a)** Stem essential oil, **(b)** Leaves essential oil, **(c)** Flowers essential oil.

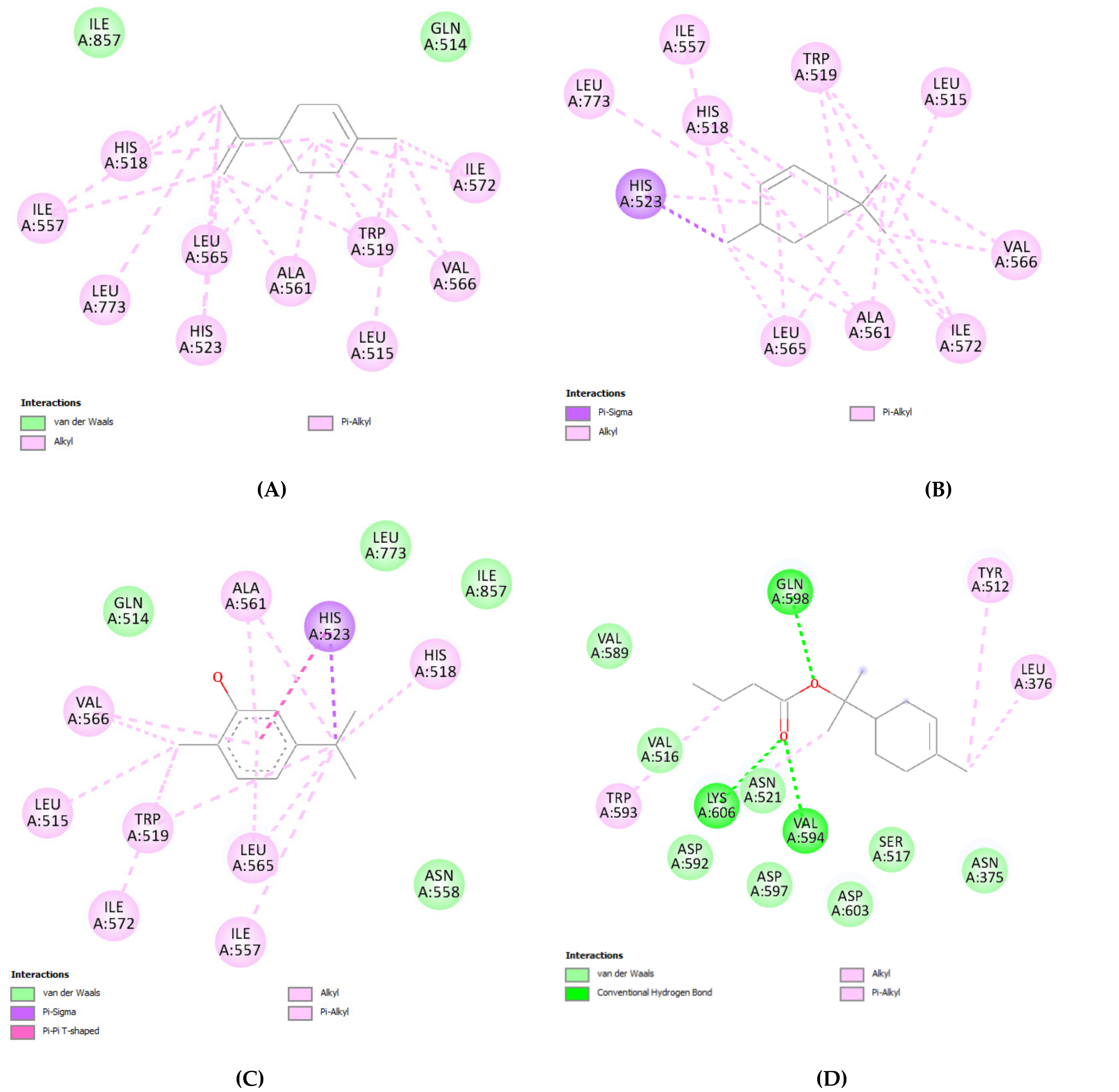
ILE A:857
GLN A:514
HIS A:518
ILE A:572
ILE A:557
LEU A:565
TRP A:519
ALA A:561
VAL A:566
LEU A:773
HIS A:523
LEU A:515
Interactions
van der Waals
Alkyl
Pi-Alkyl
(A)
ILE A:557
TRP A:519
LEU A:773
LEU A:515
HIS A:518
HIS A:523
VAL A:566
ALA A:561
LEU A:565
ILE A:572
Interactions
Pi-Sigma
Alkyl
Pi-Alkyl
(B)
LEU A:773
ILE A:857
ALA A:561
GLN A:514
HIS A:523
HIS A:518
O
VAL A:566
LEU A:515
TRP A:519
LEU A:565
ASN A:558
ILE A:572
ILE A:557
Interactions
van der Waals
Pi-Sigma
Pi-Pi T-shaped
Alkyl
Pi-Alkyl
(C)
GLN A:598
TYR A:512
VAL A:589
LEU A:376
O
O
VAL A:516
ASN A:521
TRP A:593
LYS A:606
VAL A:594
SER A:517
ASP A:592
ASN A:375
ASP A:597
ASP A:603
Interactions
van der Waals
Conventional Hydrogen Bond
Alkyl
Pi-Alkyl
(D)

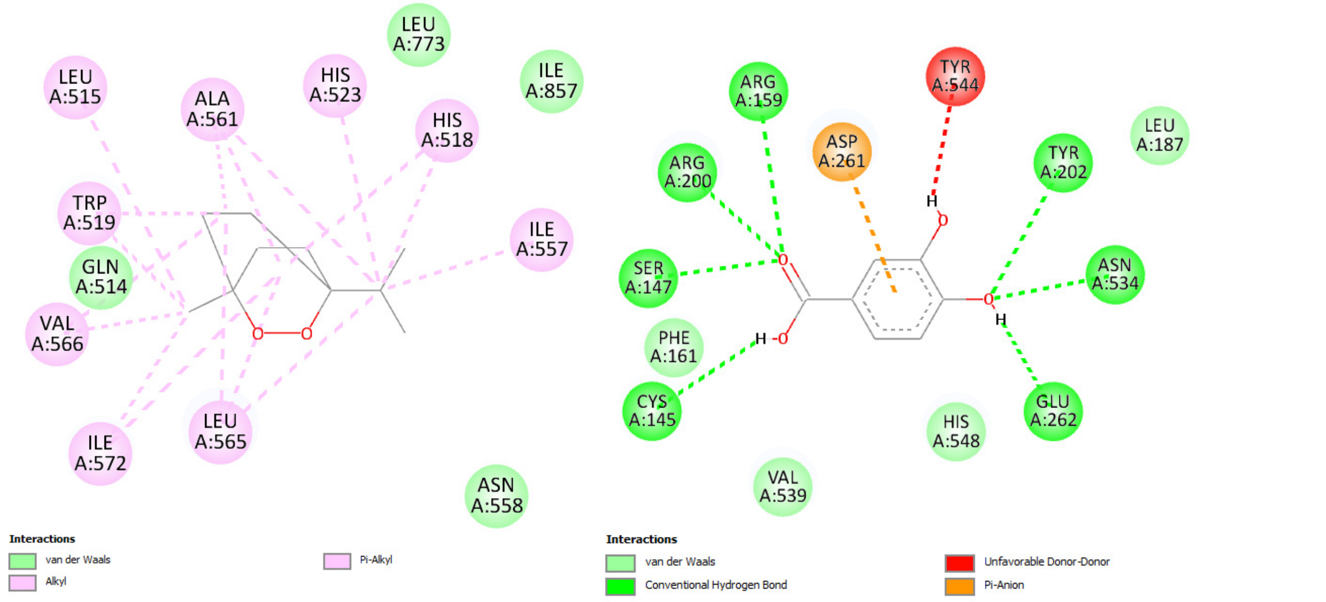

(E) **(F)**

Figure S2. 2D binding interactions of compounds D-Limonene **(A)**, 4-(+)-carene **(B)**, Carvacrol **(C)**, *trans*-β-Terpinyl butanoate **(D)**, Ascaridole **(E)**, and Protocatechuic Acid (native ligand) **(F)**, against Lipoxygenase protein **(PDB ID: 1N8Q)**

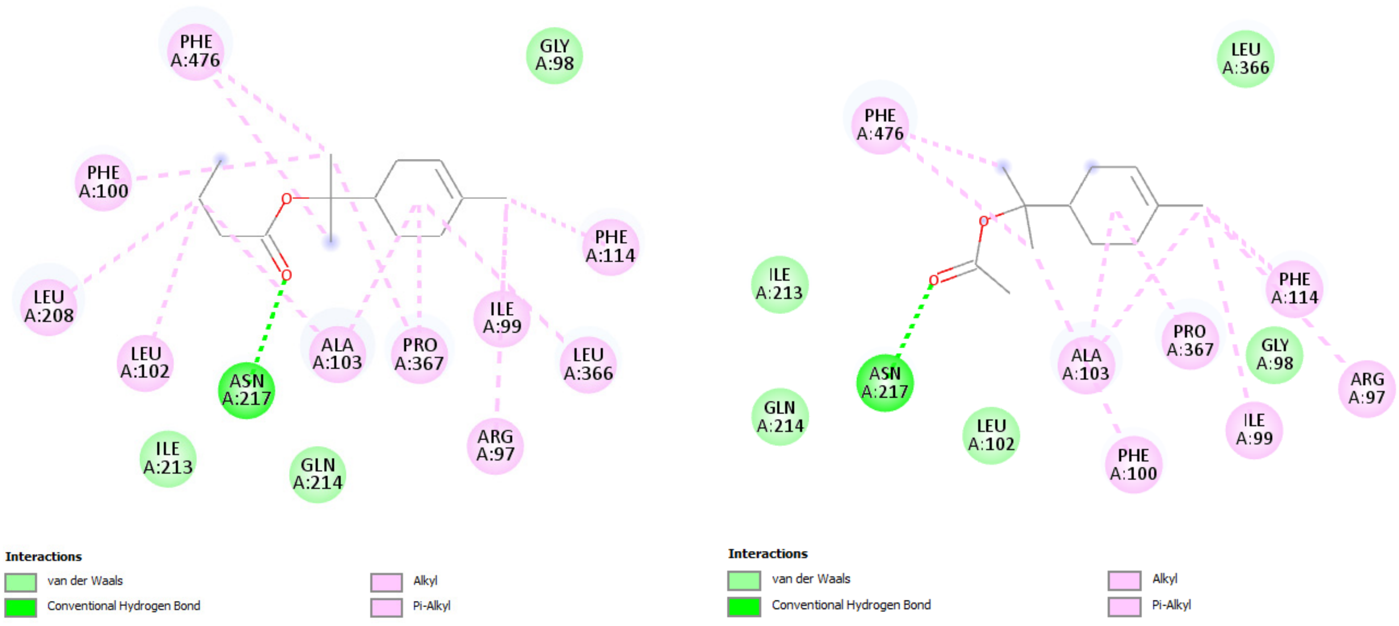

(A) **(B)**

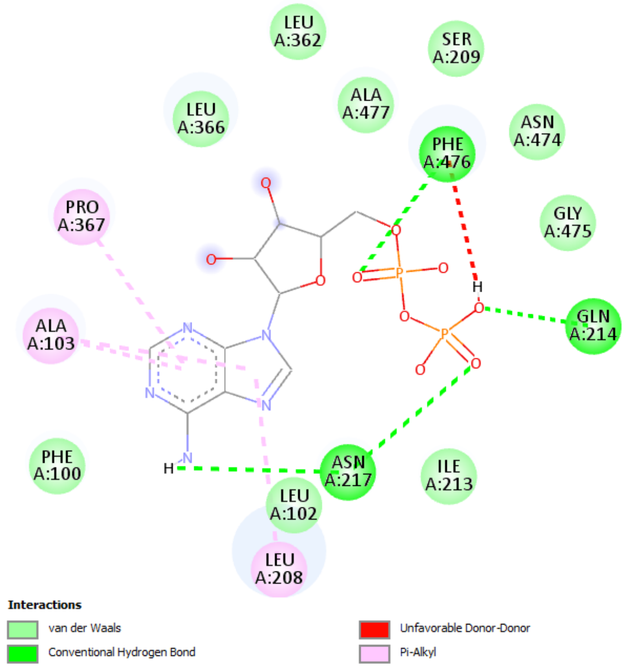

(C)

Figure S3. 2D binding interactions of compounds *trans*-β-Terpinyl butanoate **(A)**, α-Terpineol acetate **(B)**, and Warfarin (native ligand) **(C)**, against CYP450 protein **(PDB ID: 1OG5)**

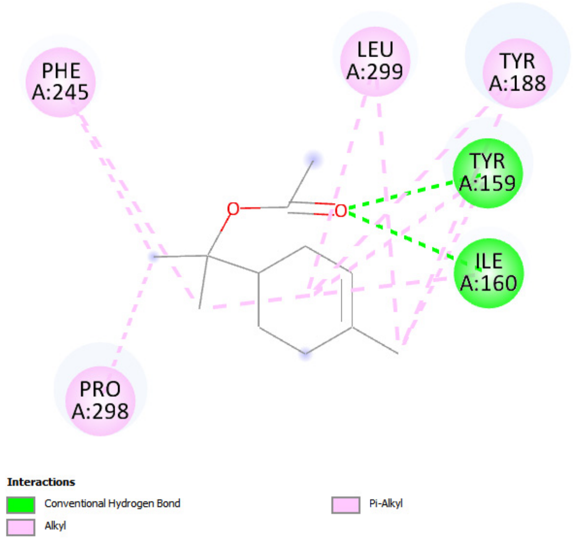

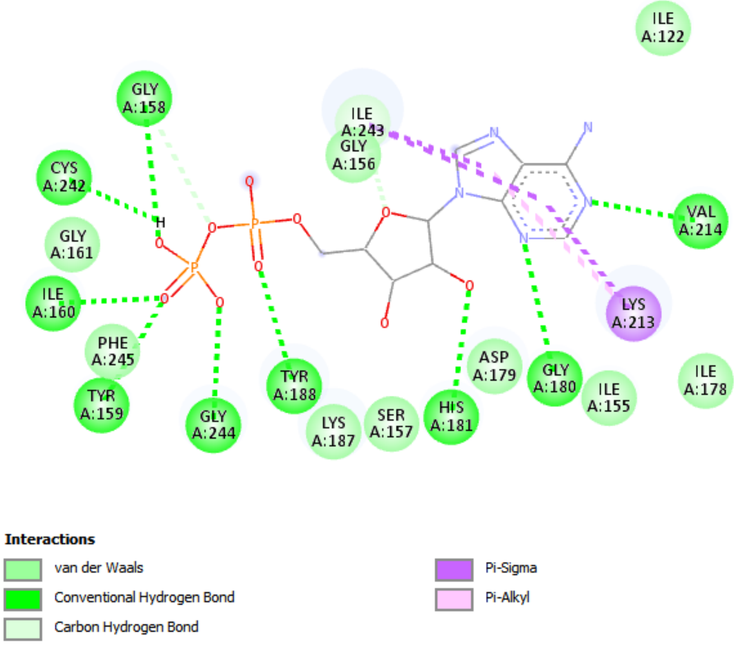

(A) **(B)**

Figure S4. 2D binding interactions of compounds α-Terpineol acetate **(A)**, and Adenosine-5'-diphosphate (native ligand) **(B)**, against NADPH Oxidase protein **(PDB ID: 2CDU)**

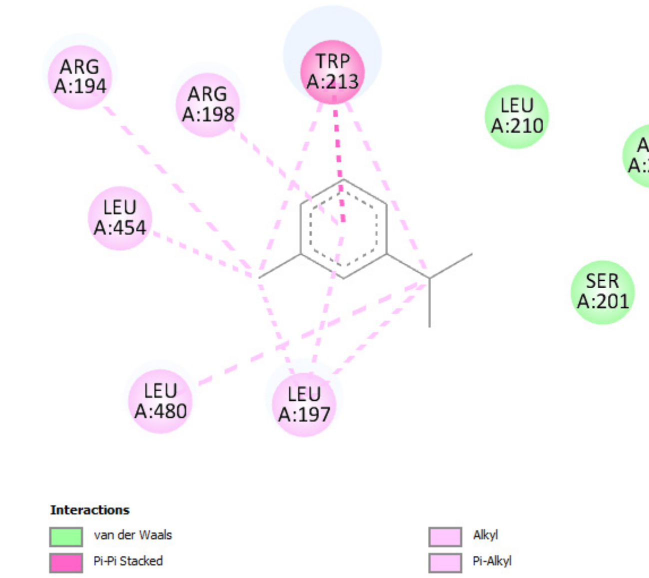
ARG A:194
ARG A:198
TRP A:213
LEU A:210
ALA A:209
LEU A:454
SER A:201
LEU A:480
LEU A:197
Interactions
van der Waals
Pi-Pi Stacked
Alkyl
Pi-Alkyl

(A)

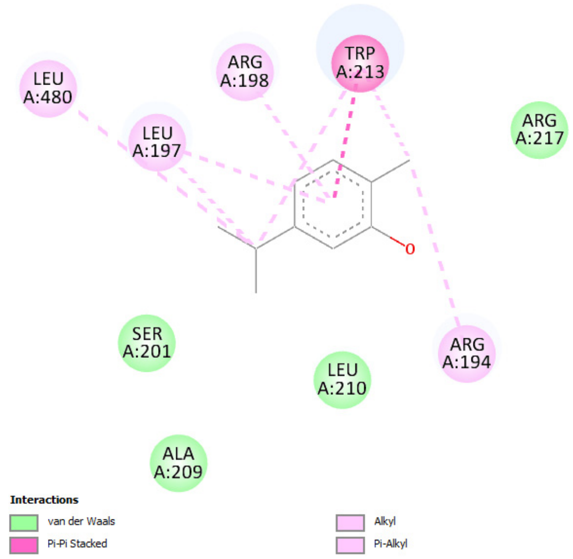
LEU A:480
ARG A:198
TRP A:213
LEU A:197
ARG A:217
O
SER A:201
LEU A:210
ARG A:194
ALA A:209
Interactions
van der Waals
Pi-Pi Stacked
Alkyl
Pi-Alkyl

(B)

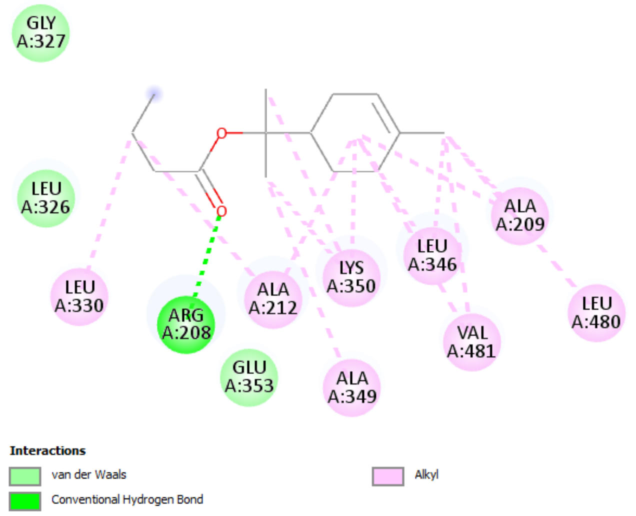
GLY A:327
O
O
LEU A:326
ALA A:209
LEU A:346
LYS A:350
LEU A:330
ALA A:212
ARG A:208
LEU A:480
VAL A:481
GLU A:353
ALA A:349
Interactions
van der Waals
Conventional Hydrogen Bond
Alkyl

(C)

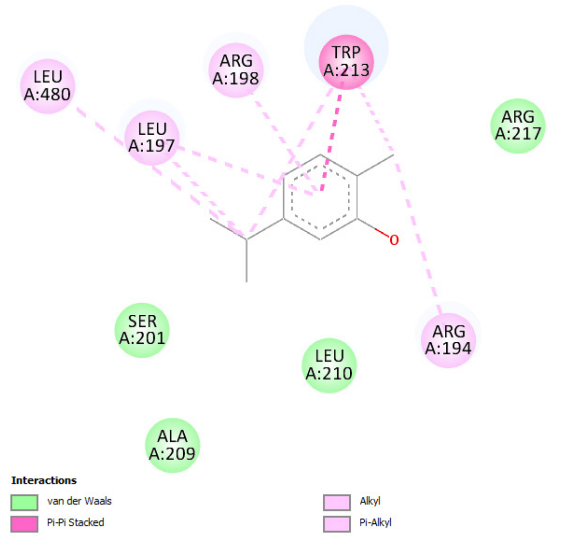
LEU A:480
ARG A:198
TRP A:213
LEU A:197
ARG A:217
O
SER A:201
LEU A:210
ARG A:194
ALA A:209
Interactions
van der Waals
Pi-Pi Stacked
Alkyl
Pi-Alkyl

(D)

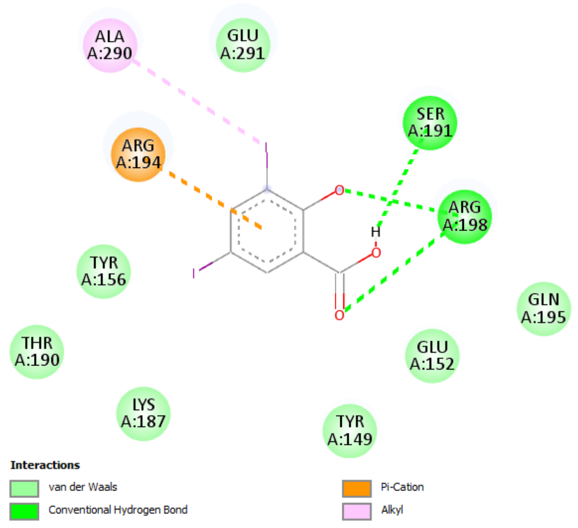

(E)

Figure S5. 2D binding interactions of the 5 most potent compounds m-Cymene **(A)**, Carvacrol **(B)**, *trans-β*-Terpinyl butanoate **(C)**, Ascaridole **(D)**, and 3,5-Diiodosalicylic Acid (native ligand) **(E)**, against Bovine Serum Albumin (BSA) protein **(PDB ID: 4JK4)**, * **All the identified components were found to be potent inhibitors of BSA protein.**

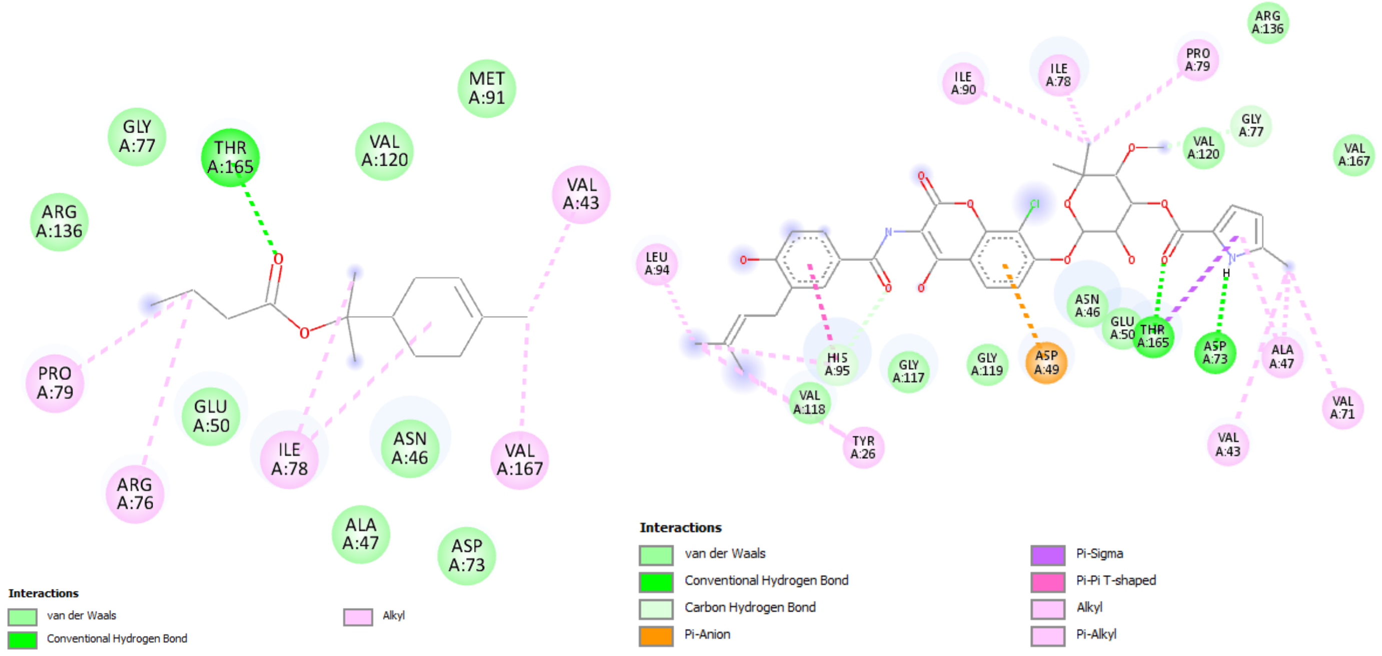

(A) **(B)**

Figure S6. 2D binding interactions of compound *trans-β*-Terpinyl butanoate **(A)**, and Clorobiocin (native ligand) **(B)**, against DNA Gyrase Topoisomerase II (*E. coli*) protein **(PDB ID: 1KZN)**.

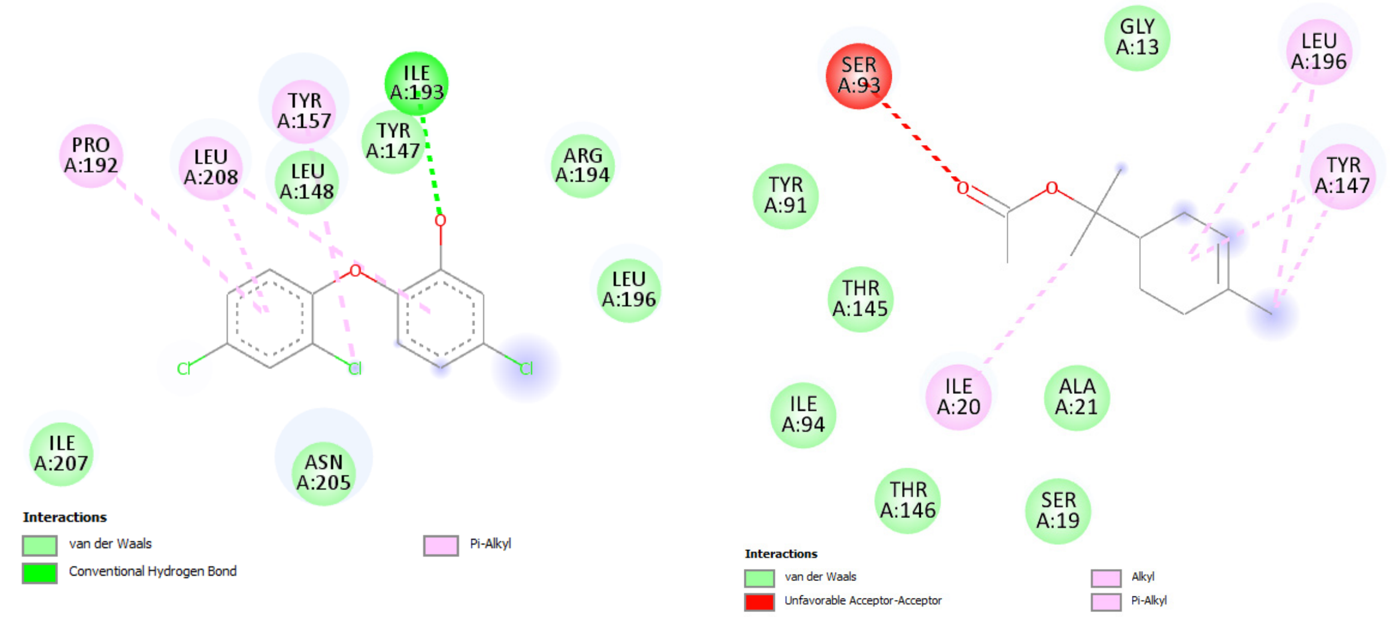

(A) **(B)**

Figure S7. 2D binding interactions of compound α-Terpineol acetate **(A)**, and Triclosan (native ligand) **(B)**, against *S. aureus* Enoyl-Acyl Carrier Protein Reductase (FabI) protein **(PDB ID: 3GNS)**.

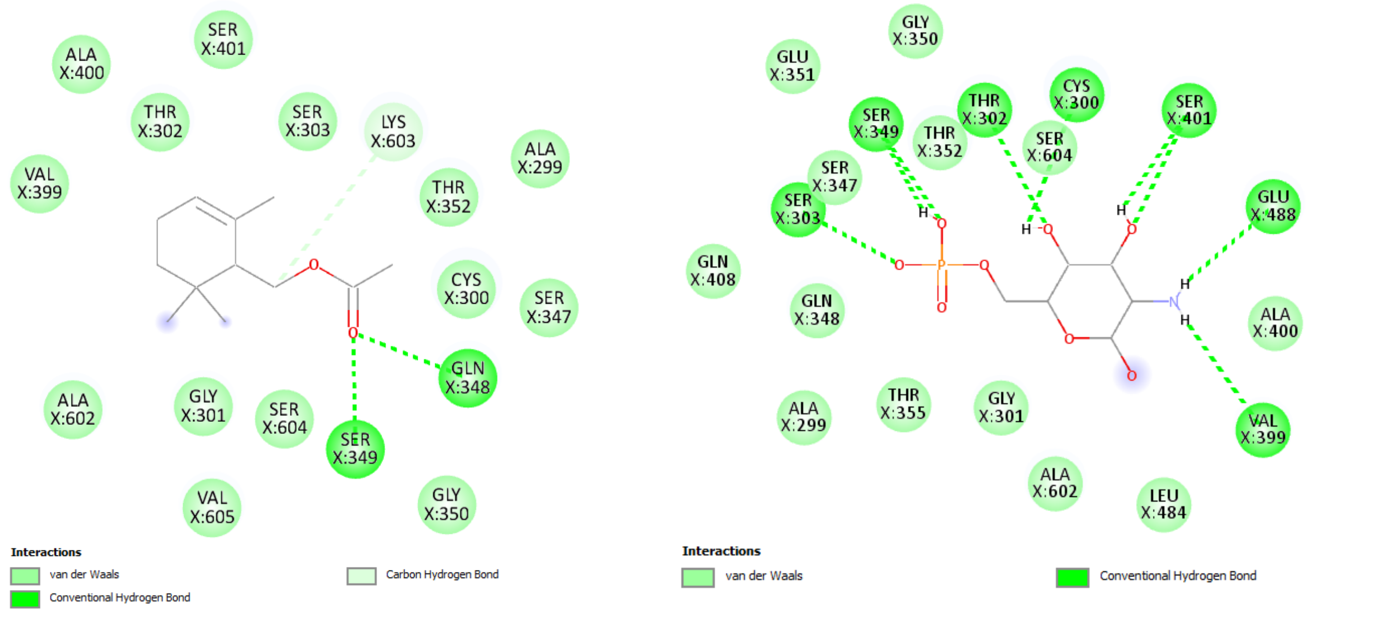

(A) **(B)**

Figure S8. 2D binding interactions of compound α-Cyclogeraniol acetate **(A)**, and Glucosamine-6- Phosphate (native ligand) **(B)**, against Glucosamine-6-Phosphate Synthase protein **(PDB ID: 2VF5)**.
